# Claudin expression during early postnatal development of the murine cochlea

**DOI:** 10.1186/s12899-018-0035-1

**Published:** 2018-01-25

**Authors:** Takayuki Kudo, Philine Wangemann, Daniel C. Marcus

**Affiliations:** Anatomy and Physiology Department, Kansas State University, 228 Coles Hall, Manhattan, KS 66506 USA

**Keywords:** Tight junctions, Inner ear, Pendrin, SLC26A4, Mouse

## Abstract

**Background:**

Claudins are major components of tight junctions, which form the paracellular barrier between the cochlear luminal and abluminal fluid compartments that supports the large transepithelial voltage difference and the large concentration differences of K^+^, Na^+^ and Ca^2+^ needed for normal cochlear function. Claudins are a family of more than 20 subtypes, but our knowledge about expression and localization of each subtype in the cochlea is limited.

**Results:**

We examined by quantitative RT-PCR the expression of the mRNA of 24 claudin isoforms in mouse cochlea during postnatal development and localized the expression in separated fractions of the cochlea. Transcripts of 21 claudin isoforms were detected at all ages, while 3 isoforms (*Cldn-16*, − *17* and − *18*) were not detected. Claudins that increased expression during development include *Cldn-9*, *− 13, − 14, − 15,* and *-19v2*, while *Cldn-6* decreased. Those that do not change expression level during postnatal development include *Cldn-1*, *− 2*, *− 3*, *− 4*, *− 5*, *− 7*, − 8, −*10v1*, −*10v2*, *− 11*, *− 12*, *−19v1*, *− 20*, *− 22, and − 23*. Our investigation revealed unique localization of some claudins. In particular, *Cldn-13* expression rapidly increases during early development and is mainly expressed in bone but only minimally in the lateral wall (including stria vascularis) and in the medial region (including the organ of Corti). No statistically significant changes in expression of Cldn-11, − 13, or − 14 were found in the cochlea of *Slc26a4*^*−/−*^ mice compared to *Slc26a4*^*+/−*^ mice.

**Conclusions:**

We demonstrated developmental patterns of claudin isoform transcript expression in the murine cochlea. Most of the claudins were associated with stria vascularis and organ of Corti, tissue fractions rich in tight junctions. However, this study suggests a novel function of *Cldn-13* in the cochlea, which may be linked to cochlear bone marrow maturation.

**Electronic supplementary material:**

The online version of this article (10.1186/s12899-018-0035-1) contains supplementary material, which is available to authorized users.

## Background

Tight junctions are structures consisting of proteins that join epithelial and endothelial cells to form continuous sheets and tubules which separate two liquid compartments. They consist of claudins [1], occludins [2] and other proteins that form a band-like network known as tight junction strands. These junctions are known to perform several functions (barrier, pore and fence), and are composed of several types of proteins: transmembrane (e.g., claudins and occludin), cytoplasmic, signaling and adapter links to the cytoskeleton [1, 3]. Barrier function refers to the restriction of paracellular movement of fluid constituents between the two fluid compartments that are separated by the cell layer. Pore function refers to the selective permeability of the paracellular barrier to those solutes that can pass between the fluid compartments. The fence function refers to the restriction of lateral movement of membrane proteins and lipids within the face of the plasma membrane, which retains the separate physiological functions of the luminal and abluminal cell membranes that are necessary to carry out vectorial transport of solutes and water.

**Fig. 1:**
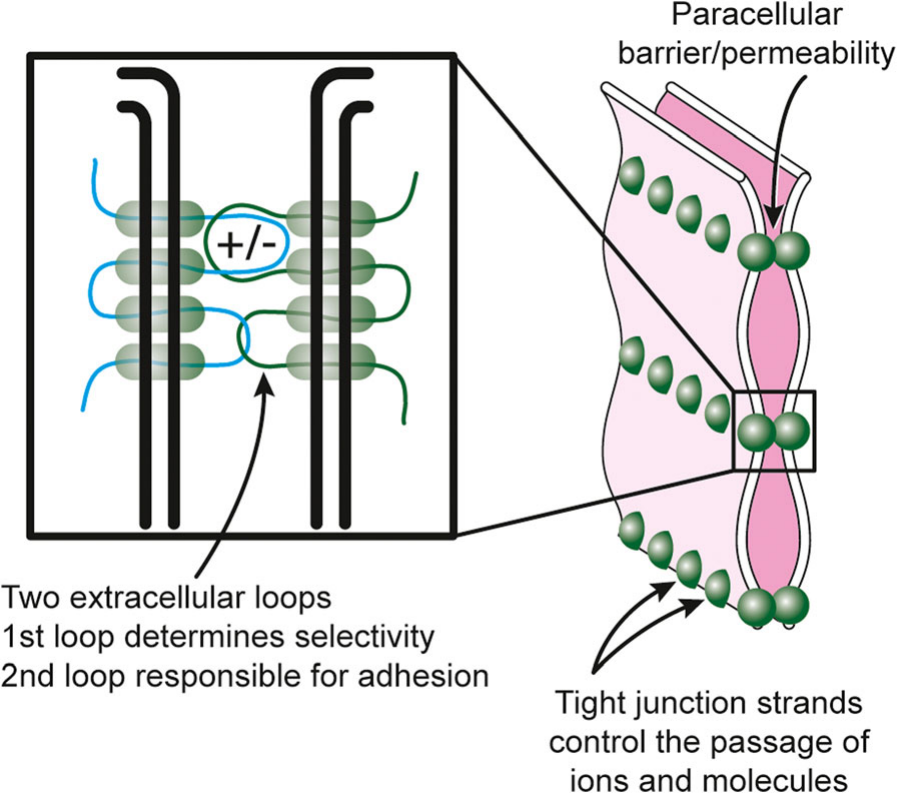
Schematic diagram of claudin structure. *Left panel*. Apposed epithelial cell membranes with one integral-membrane claudin molecule in each cell. Each claudin has four transmembrane segments including two extracellular loops and both the C-terminal and N-terminal ends within the cytoplasm. The extracellular loops of the apposed claudins associate with the respective loops of the adjacent claudin. The *right panel* depicts each claudin molecule as a sphere within the adjacent cell membranes, represented at a lower magnification than in the left panel. Ion selectivity is imparted to the tight junction by claudin amino acids with a net charge in the first extracellular loop

Claudins are a family of more than 20 subtypes [1]. The specific isoforms of claudin included in a tight junction are the primary determinant of paracellular permeability [3]. Common structures of the claudin family include four transmembrane domains and two extracellular loops (Fig. 1). It is thought that charged amino acids in the first extracellular loop define the permeability of tight junctions, while the second extracellular loop contributes toward adhesion of the apposed cell membranes (Fig. 1) [1, 4]. Multiple claudin isoforms are usually co-expressed in one tissue and their mixing ratio determines the permeability properties of the tight junction in that tissue [1, 5]. Claudins are regulated in their expression, same-cell and neighboring-cell interactions, modulations and degradation by numerous separate pathways and networks [3].

Claudins are known to be critical for normal hearing [6, 7]. A major driving force for the ionic currents underlying the cellular transduction of sound into corresponding electrical signals to hearing centers in the brain is the endocochlear potential, the transepithelial voltage across the inner ear epithelium [8]. This voltage is generated within the multi-layered stria vascularis in the cochlear lateral wall and originates as a potential difference across the basal cell layer of the stria between the intrastrial fluid space and the perilymph pervading the fibrous spiral ligament [8]. This potential difference across the basal cell layer is supported by the barrier function of the highly dense tight junctions between the basal cells, as confirmed by the reduction of endocochlear potential and the resulting deafness in adult *Cldn-11* knockout mice [6]. In contrast to this pathology of stria vascularis, mutation of *Cldn-14* led to degeneration of a different cochlear structure, the sensory organ of Corti, and was associated with the human hereditary deafness DFNB29 [9, 10]. It is to be expected that mutations of other claudin isoforms in the cochlea could lead to impaired hearing. In addition to these examples of claudin isoform localization and expression, three other groups of investigators have reported expression of claudins in the cochlea [11–13]. Kitajiri and colleagues [12] examined *Cldn-1* to *Cldn-18* using immunohistochemistry, but their study was limited by a lack of antibodies for some claudins. We localized in the current study transcript expression of most of the claudin isoforms in multiple tissue fractions of the cochlea, including the outer layer of cochlear bone.

One of the most common hereditary deafness genes is pendrin (SLC26A4), which has been surprisingly shown to exert its strongest effects on cochlear function by expression in the endolymphatic sac during early development [14]. Lack of pendrin expression was found to be accompanied by delays in cochlear bone development and in expression of other genes due to an apparent local hypothyroidism [15]. It therefore was of interest to determine whether the expression of *Cldn-13*, observed in the present study to be predominantly expressed in the outer bone fraction, would be altered by deletion of the *Slc26a4* gene in the mouse model.

The aim in the present study was to determine a) expression of claudin transcripts during early development, b) localization of the claudin isoforms among the cochlear regions and c) the potential effects of *Slc26a4* knockout on claudin isoform expression.

## Methods

*Slc26a4*^*+/−*^ and *Slc26a4*^*−/−*^ mice were obtained from a colony at Kansas State university and the heterozygous mice served as controls. Animals were deeply anesthetized with sodium pentobarbital (100 mg/kg i.p.). Temporal bones were removed from both male and female mice and whole cochleae were collected from age-sex matched littermates of *Slc26a4*^*+/−*^ and *Slc26a4*^*−/−*^. Expression of claudins was determined on RNA isolated from a) whole cochlea, b) cochlear lateral wall tissues, c) cochlear medial fraction tissues, and d) outer cochlear bone. Lateral wall tissues were further microdissected into spiral ligament and stria vascularis fractions, while the medial fraction was further microdissected into organ of Corti and modiolus fractions. All procedures involving animals were approved by the Institutional Animal Care and Use Committee of Kansas State University (protocol 2925).

Total RNA was isolated from these tissues using the RNeasy Micro Kit (Qiagen, Valencia, CA; Cat #7400) and care was taken that RNA was extracted from all cell types. Recombinant bovine DNase I, Grade 1 (Roche Diagnostics Corp, Indianapolis, IN; catalog # 04536282001) was used to remove residual DNA. The quality and quantity of 18S rRNA were determined by using the RNA 6000 Nano Kit (Agilent Technologies, Santa Clara, CA; catalog # 5067–1511) with a Bioanalyzer (Agilent Technologies; Model 2100) and a spectrophotometer (Thermo Scientific, Wilmington, DE; NanoDrop 8000). The amount of RNA in each sample was calculated as the average of the results of the Bioanalyzer and NanoDrop assays.

Primer pairs for mouse *Cldn-1* to *− 23* (excluding *Cldn-21*) were designed using software Primer3 (http://primer3.sourceforge.net/). The sequences of primers are documented in Table 1 and were validated with RNA from positive control tissues (tibia, liver, lung, intestine, kidney, stomach, skin, brain). mRNA expression was measured by quantitative RT-PCR using a Bio-Rad icycler iQ thermocycler and QuantiTect SYBR Green RT-PCR Kit (Qiagen; catalog # 204243). Claudin mRNA was normalized against 18S. The calculation method has been described previously [16]. Melting curve measurements were made with the Bio-Rad thermocycler and the PCR product size was measured by using a DNA assay (Agilent DNA 1000 Kit; catalog #5067–1504) on the Agilent Bioanalyzer to exclude the detection of nonspecific PCR products. This method yields quantitative measures of claudin isoform transcript expression that can be compared within each isoform; however, comparisons between and among isoforms are not quantitative in these experiments due to undetermined efficiencies in the RT step that vary for each primer pair [16].

**Table 1:** Primers for RT-PCR

Template (v: splice variant)	primers	sequences	product length	GenBank Accession Number
18S	18S_L	gaggttcgaagacgatcaga	316	X00686
	18S_R	tcgctccaccaactaagaac		
Claudin-1	cldn1L	cgactccttgctgaatctga	390	NM_016674
	cldn1R	cgtggtgttgggtaagaggt		
Claudin-2	cldn2L	ggtggcttctgtgaggacat	333	NM_016675
	cldn2R	ctttcccttggcttcttgtg		
Claudin-3	cldn3L	cgggagtgcttttcctgtt	344	NM_009902
	cldn3R	tgctggtagtggtgacggta		
Claudin-4	cldn4L3	ccgcgacttctacaacccta	326	NM_009903
	cldn4R3	gtccccagcaagcagttagt		
Claudin-5	cldn5L2	gaagccgtgtgtggatgac	307	NM_013805
	cldn5R2	gccctttcaggttagcaggt		
Claudin-6	cldn6L1	ctactgaggctgggaggatg	363	NM_ 018777
	cldn6R1	ttgtgtgagcagggaagtgt		
Claudin-7	cldn7L1	caactgctgggcttttcaat	329	NM_ 016887
	cldn7R1	gccttcttcgctttgtcatc		
Claudin-8	cldn8L4	agccggaatcatcttcttca	399	NM_018778
	cldn8R4	cagtgtgggctccatttctc		
Claudin-9	cldn9L2	tactccatcccttcccgttc	331	NM_ 020293
	cldn9R2	ctgaggtccaggttccagag		
Claudin-10v1	cldn10v1L2	gggatttttcggttccattt	378	NM_023878
	cldn10v1R2	tctccttctccgccttgata		
Claudin-10v2	cldn10v2L	tttttcggttccatttttgc	375	NM_ 021386
	cldn10v2R	atctccttctccgccttgat		
Claudin-11	cldn11L2	gccgaaaaatggacgaact	315	NM_008770
	cldn11R2	gggcacatacaggaaaccag		
Claudin-12	cldn12L3	cagatgtgctcctgttgcat	304	NM_022890
	cldn12R3	cccgtgtaaatcgtcaggtt		
Claudin-13	cldn13L2	tcgggaaaacaggtggatac	385	NM_020504
	cldn13R2	gttgacacagagcaggatgc		
Claudin-14	cldn14L3	ctgggcttcatctcctcatc	332	NM_ 019500
	cldn14R3	aagagcacctccttccctgt		
Claudin-15	cldn15L2	aagacggcagacaagaatcg	305	NM_021719
	cldn15R2	caaagatggtgttggtggtg		
Claudin-16	cldn16L1	gcagggaccacattactcatt	389	NM_053241
	cldn16R1	taaacggcacaggaacacag		
Claudin-17	cldn17L11	ggctgaagcagtaggccaag	314	NM_181490
	cldn17R11	tgagagcaaccaaggcaaga		
Claudin-18	cldn18L4	gaacccttccccaagaagag	355	NM_019815
		caagctggaaaatcgaccat		
Claudin-19v1	cldn19v1L	gaagggctgtggatgtcttg	321	NM_001038590
	cldn19v1R	aggagtgctggggttgaag		
Claudin-19v2	cldn19v2L2	tgctggctacatcttgtggt	306	NM_153105
	cldn19v2R2	gacagttgaatggggttgct		
Claudin-20	cldn20L2	cagctccttgctttcatcct	356	NM_001101560
	cldn20R2	aagcagactcctccagcaaa		
Claudin-22	cldn22L2	ggcttggagagacacaggag	342	NM_029383
	cldn22R2	tttctggattggcttgcttc		
Claudin-23	cldn23L2	tactacagcgacggacagca	320	NM_027998
	cldn23R2	cagttagaggaaggcgacca		

**Fig. 2:**
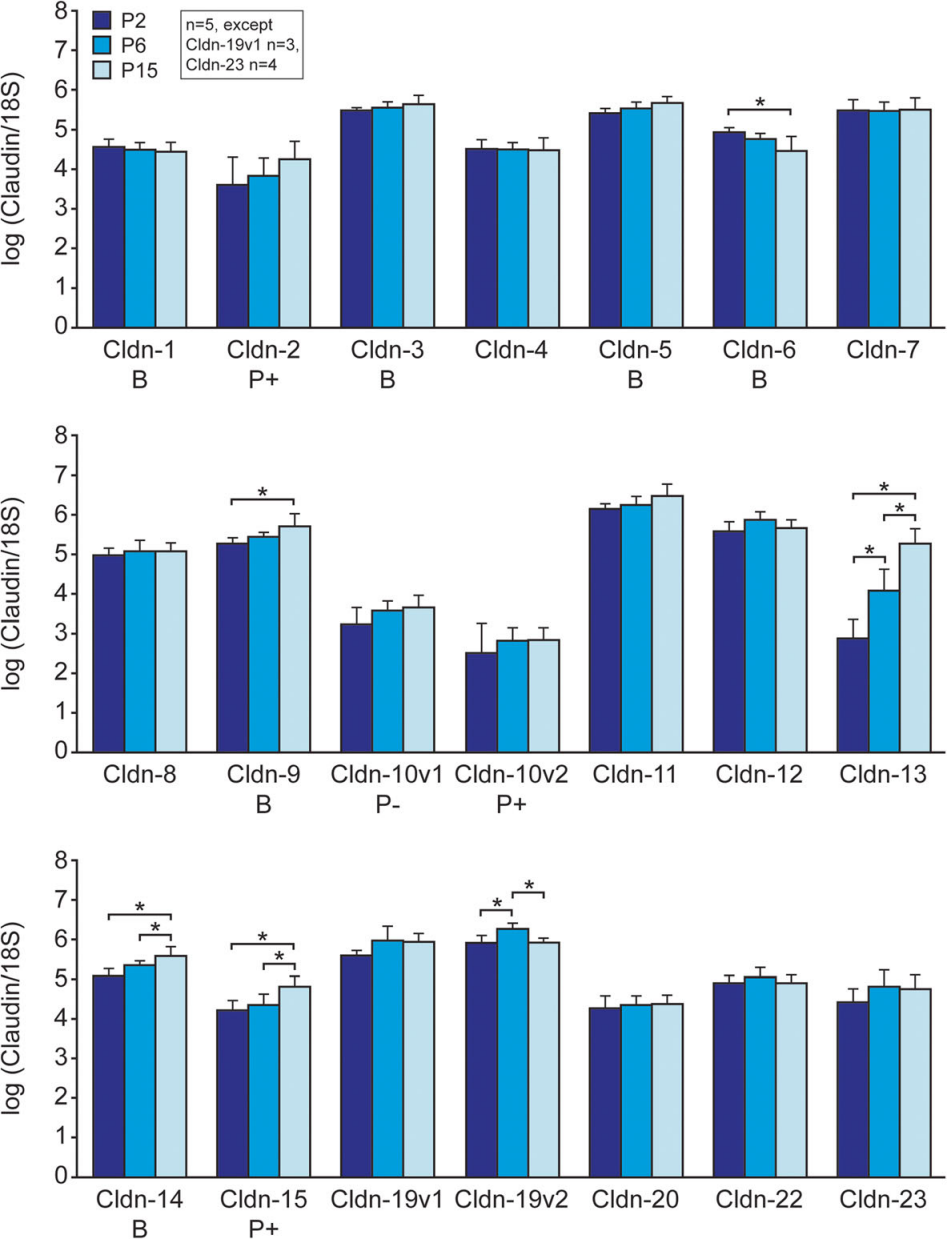
Developmental expression levels of 21 cochlear transcripts for claudin isoforms at P2, P6 and P15 in *Slc26a4*^+/−^ mice. Bars for each isoform are in chronological order, left to right; Top, middle and bottom panels are numerically-increasing isoforms. *Cldn-16*, *− 17* and *− 18* did not show detectable specific amplification. Claudins associated with established permeability properties are designated in the second row of the labels: B, barrier; P, permeable pore; +, cation-selective pore; −, anion-selective pore [17]. Asterisks indicate significant difference (*P* < 0.05) between bars embraced by brackets using one-way ANOVA and the Holm-Sidek post-test. The absence of brackets and asterisks indicates differences are not significant . Error bars, Standard Deviation. The individual descriptive statistics are derived from n cochleae, as indicated on the graph

**Fig. 3:**
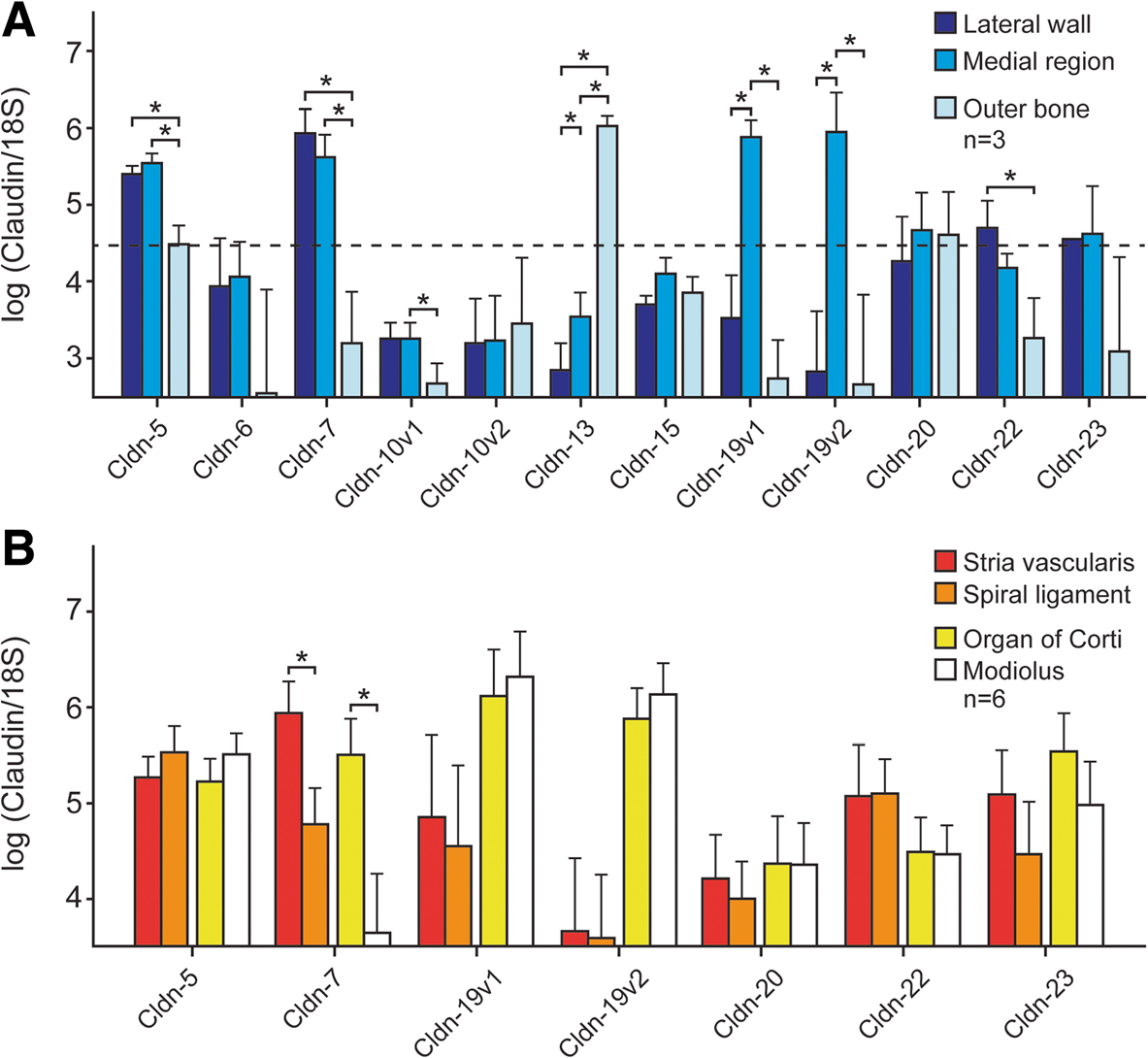
Localization of selected claudin isoforms (see text) in the cochlea. **a** The cochlea was dissected into three parts (lateral wall, medial region and outer bone). Transcript expression is shown for *cldn-5, − 6, − 7, −10v1, −10v2, − 13, − 15, −19v1, −19v2, − 20, − 22, − 23* (*n* = 3) in each fraction. Ages of samples are between P18 and P32 (days), mean: 22.6. Asterisks indicate significant difference between tissues. Claudins in the lateral wall and medial fractions that were highly expressed (claudin mRNA/18S > 4.5; dashed line) in this experimental series were analyzed in the more-finely separated tissues of the following series. **b** The lateral and medial fractions were each subdivided into two smaller fractions in order to obtain finer resolution of location (*n* = 6). Lateral wall: stria vascularis and spiral ligament; Medial region: organ of Corti and modiolus. Ages of samples are between P19 and P28 (days), mean: 22.0. Asterisks indicate significant difference (P < 0.05) using the two-way analysis of variance as indicated by brackets; *, significant. Non-significant comparisons are not shown and comparisons other than stria vascularis – spiral ligament and organ of Corti – modiolus are given in the Additional file 1. Error bars, Standard Deviation

**Fig. 4:**
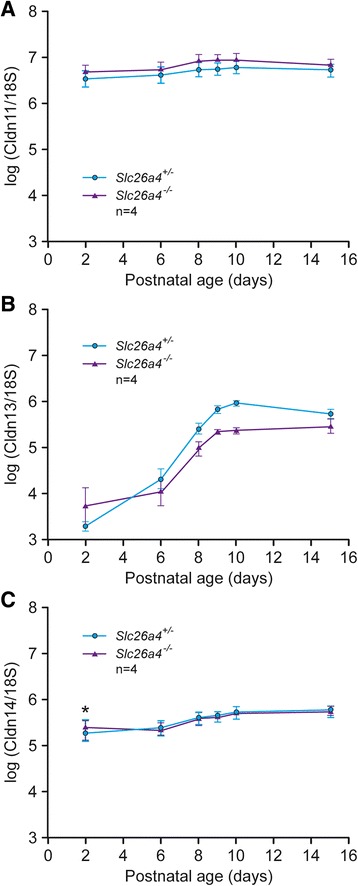
Developmental effects of *Slc26a4* gene knockout on expression in the whole cochlea of known hearing-related claudins, cldn-11, − 13, − 14, between postnatal ages 2–16 days. Blue circles represent *Slc26a4*^+/−^ and red triangles represent *Slc26a4*^−/−^ (*N* = 4 each). **a** *Cldn-11*; (**b**) *Cldn-13*; (**c**) *Cldn-14*. The analyses showed no statistically significant interaction between age and genotype in all three genes and no further comparisons of individual paired genotypes were made. Error bars are Standard Error of the Mean. The individual descriptive statistics are derived from *n* = 4 cochleae of each genotype

Data are given as means ± standard deviation (SD) or ± standard error of the mean (SEM), as reported in Results. N values refer to the number of cochleae (Figs. 2 and 4) or to the number of isolated tissues (Fig. 3a and b) which are the same as the numbers of RT-PCR reactions analyzed. A one-way analysis of variance (ANOVA) with Holm-Sidak method post-test (Figs. 2 and 3) or a two-way ANOVA which tested for statistical significance of interaction between age and genotype. Since no statistically significant interaction was found for the 3 transcripts tested in the experiments shown in Fig. 4, individual paired differences were not assessed. *P* values of < 0.05 were considered as significant differences; analyses were performed with SigmaStat for Windows Version 4.0 software.

## Results and discussion

We first determined the transcript expression level of 24 claudin isoforms in the whole cochlea of normal (*Slc26a4*^*+/−*^) mice at three different ages after birth: P2 and P6 before the onset of endocochlear potential generation and hearing in mice, and at P15, after acquisition of hearing. Transcripts of 21 claudin isoforms were detected at all ages, while 3 isoforms (*Cldn-16*, − *17* and − *18*) were not detected (Fig. 2). The permeability properties of several isoforms have been unambiguously determined [17, 18] and are shown in (Fig. 2), as described in the figure legend. *Cldn-10* [19] and *Cldn-19* were each determined for two splice variants, v1 and v2 (Table 1).

Six cochlear claudin isoforms increase with development at P6 and/or P15: *Cldn-9*, *− 13, − 14, − 15,* and *-19v2*. By contrast, *Cldn-6* expression decreases with development. Cochlear claudins that do not change significantly with development include: *Cldn-1*, *− 2*, *− 3*, *− 4*, *− 5*, *− 7*, − 8, −*10v1*, −*10v2*, *− 11*, *− 12*, *−19v1*, *− 20*, *− 22, and − 23*. As described above, endocochlear potential normally develops between P6 and P15. So genes that change their expression in this period might be involved in establishment of the special properties of the paracellular barrier of the epithelial cells that border the endolymph, and thereby provide the resistive barrier that supports the large endocochlear potential. *Cldn-19v2* appeared to increase expression only transiently during this period. The post-natal changes in expression of multiple claudin isoforms are consistent with the likely presence of factors that regulate claudin expression during development. Most striking of all, *Cldn-13* shows a remarkably large increase in cochlear expression compared to the others. Previously, Abuazza et al. [20] reported maturational decrease of *Cldn-6, − 9* and *− 13* transcripts and of paracellular protein in several segments of the mouse kidney. They suggested these changes may contribute to developmental changes in the paracellular permeability of kidney tubules. In our study of the cochlea, *Cldn-6* undergoes developmental *decrease* in transcript expression from P2 to P6, and further from P6 to P15, as in the kidney (Fig. 2). By contrast to the kidney, *Cldn-9* and -*13* transcripts *increased* from P2 to P6, and further from P6 to P15 (Fig. 2).

The cochlear tissues expressing these claudins were resolved in two subsequent experimental series. In the first series (Fig. 3a), cochleae of adult (P18-P32, mean P22.6) *Slc26a4*^*+/−*^ mice were subdivided into three fractions: 1) lateral wall (exclusive of outer bone), 2) medial region, and 3) outer bone. These fractions were assayed for 12 claudin isoforms: *Cldn-5*, − *6*, − *7*, −*10v1,* −*10v2,* − *13, − 15, −19v1, −19v2, − 20, − 22*, and − *23.* All of these claudins were detected both in the lateral wall and the medial region fraction. *Cldn-19v1* and *Cldn-19v2* were expressed most strongly in the medial region.

Interestingly, *Cldn*-*13* was expressed virtually exclusively in the outer bone fraction, in spite of the statistically significant difference in the minimal expression in the two soft tissues. Wongdee et al. examined claudin expression in skull and tibia bone [21] and determined localization of *Cldn-5, − 11, − 14, − 15* and − *16*. The expression was limited to the cells lining the bone (periostieum), suggesting a function of claudin other than tight junction formation. They, however, did not test bone for the presence of *Cldn-13*. Johnson et al. reported *Cldn-13* expression in G1E cells, a proerythroblastic cell line [22] and *Cldn-13* was identified in a stress induced erythropoiesis pathway that is mainly expressed in tissues associated with haematopoietic function [23]. It is therefore likely that expression of *Cldn-13* in cochlear outer bone might originate from the associated bone marrow, which develops during the early postnatal period [15, 24]. In support of this proposition, it was found that *Slc26a4*^*−/−*^ mice exhibit delayed bone marrow maturation between P6 and P15 [24]. Mouse *Cldn-13* does not have a human homolog [3].

In the second series, cochleae of adult (P19-P28, mean P22.0) *Slc26a4*^*+/−*^ mice were subdivided further into four micro-dissected fractions: 1) the stria vascularis and 2) spiral ligament fractions were separated from the lateral wall; 3) the organ of Corti and the 4) modiolus were separated from the medial structures. Claudins in the lateral wall and medial fractions that gave high expression signals (claudin mRNA/18S > 4.5) in the first experimental series (Fig. 3a) were analyzed in the more-finely separated tissues of the second series (Fig. 3b). The epithelial fractions (stria vascularis and organ of Corti) were found to express *Cldn*-7 more strongly than their respective primarily non-epithelial fractions, spiral ligament (fibrocytes) and modiolus (neurons). By contrast, the other six isoforms did not show statistically significant differences between the epithelial fractions and their respective adjacent non-epithelial fractions. Non-significant comparisons are not shown in Fig. 3b and comparisons other than stria vascularis – spiral ligament and organ of Corti – modiolus are given in the Additional file 1.

Three claudins were selected to investigate the possible effect of *Slc26a4* gene deletion on inner ear developmental expression of claudins. Developmental expression of the three isoforms demonstrated a dramatic postnatal increase in Cldn-13 that was not characteristic of the other two claudins, consistent with the notion that Cldn-13 is not regulated by a mechanism common to the claudins highly expressed in the epithelial tissues. We examined RNA from whole cochleae from age- and sex-matched littermates of *Slc26a4*^*+/−*^ and *Slc26a4*^*−/−*^ and analyzed by two-way ANOVA 1) *Cldn-11*, which is expressed in basal cells of stria vascularis [12], and whose deletion in mice causes hearing loss, 2) *Cldn-13*, which is expressed in cochlear outer bone (this report), and 3) *Cldn-14*, which is expressed in organ of Corti and is responsible for human hereditary deafness DFNB29. The results of analysis (Fig. 4) showed no statistically significant interaction between age and genotype in all three genes and no further comparisons of individual paired genotypes were made.

“*Cldn-21*” was not included in this study. The nomenclature has varied and developed since 2001 and was not identified in mouse at the time of this study [25]. The mouse gene currently accepted as *Cldn-21* [25, 26] has been heterologously expressed in MDCK epithelial cell cultures, immunolocalized to sites that also express the tight-junction protein occludin, and was shown to participate in a Na^+^-selective paracellular transport pathway [26].

Some of our data differ from previous observations: expression of *Cldn-5*, *− 6* and *− 15* was not detected by Kitajiri et al. [12], but were observed in our experiments. In kidney, *Cldn-5* and *-15* are expressed in endothelial cells, not epithelial cells [27]. By contrast, Kitajiri et al. [12] reported that there had been no expression of *Cldn-5* and *Cldn-15* in stria vascularis nor spiral ligament, both highly vascularized tissues. We found *Cldn-6* expression, but it gradually decreased during early development. Consistent with our observation, Kitajiri et al. [12] did not see any expression of *Cldn-6* in the adult cochlea.

## Conclusions

We analyzed 24 claudins in structures of the inner ear. Previous studies did not show the presence and localization of *Cldn-7, Cldn-13, Cldn-19* to *− 23* in the cochlea, but the results of our study showed regional localization of transcripts of these genes in the cochlea and developmental changes in two of them. We observed that *Cldn-13* is expressed in bone and that its expression increased rapidly during early postnatal development. Most of the claudins were expressed in stria vascularis and organ of Corti, tissue fractions rich in tight junctions. However, this study suggests a novel function of *Cldn-13* in the cochlea, which may be linked to cochlear bone marrow maturation.

## Additional file

Additional file 1: “Claudin expression raw data”. This file contains the data points collected and analyzed in the text and figures. (XLSX 86 kb)

## Abbreviations

*Slc26a4*^−/−^: *Slc26a4* gene knockout mouse
*Slc26a4*^+/−^: *Slc26a4* heterogeneous mouse
